# Nasal DNA methylation differentiates corticosteroid treatment response in pediatric asthma: A pilot study

**DOI:** 10.1371/journal.pone.0186150

**Published:** 2017-10-13

**Authors:** Xue Zhang, Jocelyn M. Biagini Myers, Veda K. Yadagiri, Ashley Ulm, Xiaoting Chen, Matthew T. Weirauch, Gurjit K. Khurana Hershey, Hong Ji

**Affiliations:** 1 Division of Human Genetics, Cincinnati Children’s Hospital Medical Center, Cincinnati, Ohio, United States of America; 2 Pyrosequencing lab for genomic and epigenomic research, Cincinnati Children’s Hospital Medical Center, Cincinnati, Ohio, United States of America; 3 Division of Asthma Research, Cincinnati Children’s Hospital Medical Center, Cincinnati, Ohio, United States of America; 4 Department of Pediatrics, University of Cincinnati College of Medicine, Cincinnati, Ohio, United States of America; 5 Center for Autoimmune Genomics and Etiology, Cincinnati Children’s Hospital Medical Center, Cincinnati, Ohio, United States of America; 6 Divisions of Biomedical Informatics and Developmental Biology, Cincinnati Children’s Hospital Medical Center, Cincinnati, Ohio, United States of America; University of Calcutta, INDIA

## Abstract

**Background:**

Treatment response to systemic corticosteroid in asthmatic children is heterogeneous and may be mediated by epigenetic mechanism(s). We aim to identify DNA methylation (DNAm) changes responsive to steroid, and DNAm biomarkers that distinguish treatment response.

**Materials and methods:**

We followed 33 children (ages 5–18) presenting to the Emergency Department (ED) for asthma exacerbation. Based on whether they met discharge criteria in ≤24 hours, participants were grouped into good and poor responders to steroid treatment. Nasal samples were collected upon presentation to the ED (T_0_) and 18–24 hours later (T_1_). Genome-wide DNAm was measured for both time points in 20 subjects, and compared between T_0_ and T_1_ in good and poor responders respectively. DNAm at T_1_ was also compared between two responder groups. DNAm of selected CpGs was verified in the complete cohort, and expression of associated genes was examined. Interactions between DNAm, common single nucleotide polymorphism (SNP) located at the CpG sites and treatment responses were assessed.

**Results:**

Three CpGs located in the *OTX2* promoter showed responder-specific DNAm changes from T_0_ to T_1_, in which DNAm decreased in good but not in poor responders. Good and poor responders showed differential DNAm at T_1_ in 127 CpGs without and 182 CpGs with common SNP co-localization. Negative correlations between DNAm and gene expression were observed at CpGs located within the *LDHC* promoter, suggesting an impact of DNAm on gene regulation. Interactions between SNPs, DNAm and treatment response were detected.

**Conclusion:**

Acute systemic steroid treatment modifies nasal DNAm in good responders. Nasal DNAm, dependent or independent of SNPs, can differentiate response to treatment in acute asthmatic children.

## Introduction

Asthma is the leading cause of emergency care and hospitalization in children. The current standard and most effective treatment for treating asthma exacerbation is systemic corticosteroid treatment. However, due to heterogeneity in asthma phenotypes and natural history [1, 2], 40–70% of asthma treatment showed absent or incomplete efficacy [3]. Heterogeneous responses to current standard treatment regimens have been observed in children hospitalized for acute asthma exacerbations, and nearly two thirds of children who currently have asthma reported at least one attack in the previous 12 months. One potential explanation could be the suboptimal management of asthma in the childhood age group [4]. Care practices other than the standard treatments may improve asthma management; however, effective predictions of responses to standard treatments are needed in prior.

Biomarkers that may be associated with differential responses to asthma treatments have been researched in children presenting to the ED and hospitalized for exacerbation. As asthma symptoms and responses to the standard treatment can change from episode to episode in the same person, biomarkers that are more dynamic have drawn attention such as gene expression [5]. Recently, DNA methylation has been increasingly explored due to its important role in the regulation of gene expression. DNA methylation (DNAm) is the modification of cytosine by adding a methyl group to the 5’ position of C. DNAm mostly occurs in the context of CpG dinucleotides and represents an important epigenetic mechanism that regulates gene expression [6]. Several studies have successfully identified DNAm of certain nucleotides as a biomarker for asthma [7–12], and suggested that nasal DNAm marks may be useful biomarkers of disease severity and treatment response [12]. Efforts have also been made to identify DNAm markers for asthma that develops in childhood and persists into early adulthood [13] and markers for temporal asthma transition [14]. Interactions between CpG methylation and genetic variation, especially single nucleotide polymorphisms (SNPs) located at C or G that can disrupt or create a CpG site (meSNPs or CpG-SNPs), are prevalent in the human genome (~15%) [15, 16]; such interactions have been associated with several diseases including childhood asthma [17–21]. In the present study, we examined genome-wide DNAm using nasal epithelial cells to comprehensively search for CpG sites that undergo differential methylation changes in response to systemic corticosteroid treatment in good and poor steroid responder groups, as well as to identify biomarkers for treatment response. We further validated these findings by bisulfite pyrosequencing of a larger population, followed by determination of the impact of DNAm on candidate gene expression and the impact of common meSNPs on DNAm. We also evaluated associations between DNAm-modifying meSNPs (known as methylation quantitative trait locus or meQTLs) and treatment response. Our results provide novel epigenetic biomarkers for treatment response to systemic steroid in asthmatic children and potential therapeutic targets for alternative asthma therapies.

## Materials and methods

### Study population

This study was approved by the Institutional Review Board at Cincinnati Children’s Hospital Medical Center (CCHMC # 2008–1384). Using inclusion and exclusion criteria previously described [5], children aged 5–18 years who presented to the CCHMC ED with acute asthma exacerbation and had an asthma diagnosis were recruited during March 2011-Oct 2014 and written informed consents were obtained from parents or guardians of the minors. Information collected includes demographics, environmental exposures, asthma trigger information, and personal and family allergy and asthma symptom history. Parent(s)/legal authorized representatives also provided current inhaled corticosteroid (ICS) controller medication (Asmanex^®^, Flovent^®^, Qvar^®^, Pulmicort^®^, Advair^®^, Dulera^®^, Symbicort^®^, etc.). Usage of nasal steroids in the past 4 weeks was recorded. To assess baseline asthma symptom severity and control, a respiratory symptom score was calculated (based on frequency of wheeze, cough, shortness of breath, and chest tightness) [22] and the age-specific Asthma Control Test^™^ (ACT) score was collected [23]. Information regarding the hospital course was extracted from medical charts. Enrolled patients were treated according to the CCHMC evidence-based treatment protocol for inpatient asthma exacerbation and discharged as previously defined [5]. As previously described [5], they were classified as good and poor responders based on the time required for them to clinically improve to the point where they achieve discharge criteria (>24hrs or ≤24hrs).

### Treatment protocol and definition of length of stay

Enrolled patients were treated according to the CCHMC evidence-based treatment protocol for inpatient asthma exacerbation and discharged as previously defined [5]. Briefly, all patients received 2mg/kg/day of prednisone while hospitalized and ICS were continued via mouthpiece. Clinical discharge criteria were met when: (1) oxygen saturations were greater than or equal to 91% on room air for at least 6 hours; (2) no evidence of respiratory distress; and (3) reached a time point at which the patient demonstrated sufficient clinical improvement while receiving albuterol no more frequently than every 4 hours for a period of 8 hours (q4h x 2). Length of stay (LOS) was calculated as the time the disposition was set to admit to the time the subject met the clinical criteria for discharge. Based on our previous data [5], good responders were defined as those with LOS≤24 hours (short LOS) and poor responders as those with LOS>24 hours (long LOS).

### Nasal sample collection and DNA/RNA extraction

Nasal epithelial samples were collected at two time points from each subject: (1) upon presentation to the ED (T_0_) and (2) on the inpatient floor 18–24 hours after receiving systemic corticosteroids in the ED (T_1_). Nasal mucosa sampling was performed with a CytoSoft Brush (Medical Packaging Corp, CA, USA), and the samples were immediately taken to the laboratory for processing. Nasal samples collected contained >90% epithelial cells, similar to our previous findings [24]. DNA and RNA were extracted with the AllPrep DNA/RNA Micro kit (Qiagen, Hilden, Germany), according to the manufacturer’s protocols.

### Illumina 450K array processing

For genome wide DNAm assay, we used 40 samples from 20 subjects as the discovery population. Genomic DNA from the nasal cells was bisulfite treated and assayed by the Illumina Infinium HumanMethylation450 BeadChip (Illumina). Quality of the array was assessed using sample-independent and dependent internal control probes included on the array for staining, extension, hybridization, specificity and bisulfite conversion. All the samples passed the QC and were included in the discovery analysis. The signal intensities were then background-adjusted using out-of-band probes (noob), and normalized using functional normalization with R package “RnBeads”. Beta values were calculated as $\frac{signal_{methylation}}{signal{\;}_{methylation}+\;signal_{unmethylation}}$ and used in further analyses. CpG sites that were not detected in all samples at p = 0.01 level, and CpG sites on the X and Y chromosomes were excluded.

Array data have been deposited to NCBI GEO (https://www.ncbi.nlm.nih.gov/geo/) with the accession number GSE104087.

### 450K array SNP annotation and GREAT annotation

To examine whether reported sites from Illumina 450K Arrays overlap with any known polymorphisms, common SNPs from dbSNP 142 table with minor allele frequency (MAF) ≥1% were extracted and used to annotate these sites. The majority of the reported array sites do not contain strand-specific information and are CpG dinucleotides. These reported 1bp site along with its consecutive downstream base were examined for overlapping with any known common SNPs. A small portion of the reported array sites have strand-specific information and are non-CpG methylation sites (CHG, H = A, C, T). For these sites, only the reported 1bp site was examined. When a site is found to be overlapping with polymorphisms, a binary indicator along with allele information, rsID, and observed strand, are added to the reported array site as annotations. In addition to the Illumina annotation for CpG sites on arrays, we also utilized Genomic Regions Enrichment of Annotations Tool (GREAT) to search for genes in close proximity with CpG sites [25].

### Bisulfite pyrosequencing

For DNAm measurement, a total of 200ng genomic DNA was subjected to sodium bisulfite treatment and purified using the EZ DNA methylation-Gold Kit (Zymo research, Irvine, CA, USA) according to the manufacturer’s specifications. Pyrosequencing was carried out using Pyro Gold reagents with a PyroMark vacuum prep workstation and a PyroMark Q96 MD instrument (Qiagen, Valencia, CA, USA) following the manufacturer’s instructions. The generated pyrograms were automatically analyzed using Pyro Q-CpG methylation analysis software (Qiagen, Valencia, CA, USA). 100% methylation control (*Sss*I-treated human genomic DNA) and 0% methylation control (human genomic DNA amplified by GenomePlex^®^ Complete WGA kit (Sigma, St. Louis, MO, USA)) were used in validating all assays. For SNP analysis, DNA was subjected to PCR amplification without bisulfite treatment. The allele frequency of possible SNPs was calculated by the PyroMark MD 1.0 (Qiagen). Pyrosequencing assay design and genomic coordinates are as documented in S1 Table.

### Reverse transcription quantitative PCR (RT-qPCR)

Total RNA was reverse-transcribed to cDNA using the Superscript III kit (Life Technologies, NY, USA) using random hexamers according to manufacturer’s instructions. Real-time quantitative PCR was performed using the SYBR Green Master Kit and LightCycler^®^ 480 instrument (Roche Diagnostics, Indianapolis, IN, USA). PCR was carried out in triplicate from each fraction and the mean Ct value of the triplicate reaction was normalized against the mean Ct value of *GAPDH*. Primer sequences are described in S2 Table.

### Ingenuity pathway analysis

To better understand the biological meaning of the methylation changes, genes associated with identified differentially methylated CpG sites were extracted from 450K array annotation file and was imported into Ingenuity Pathway Analysis (IPA, Ingenuity Systems, Redwood City, CA) for pathway mapping, gene network detection, and upstream regulator identification. A cutoff of 0.05 was used for statistical significance in IPA analysis.

### Statistical analysis

**Fig 1:**
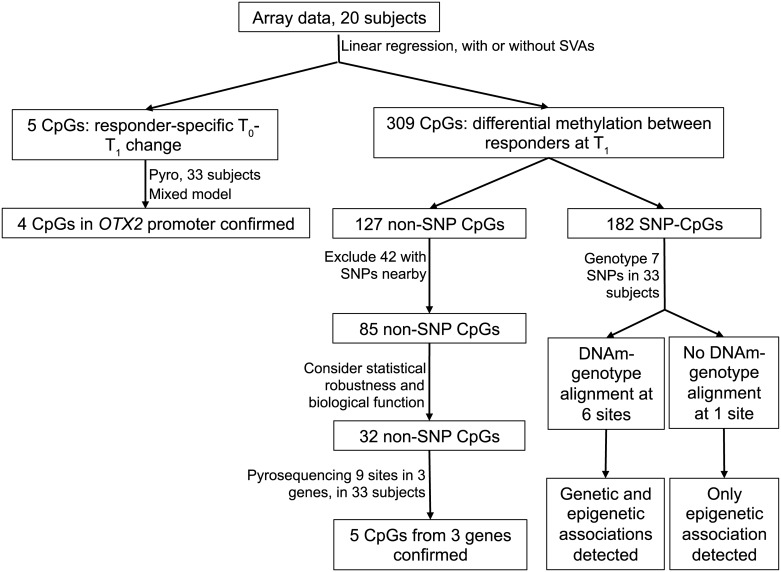
Analysis flowchart.

The analyses were performed as outlined in Fig 1. To identify CpG sites with responder-specific T_0_-T_1_ changes, the association between DNAm (beta value from the array; see detailed description in the supplementary information) and time were tested using linear regression in poor and good responders respectively. To identify CpG sites with differential DNAm between poor and good responders, we tested the association between beta value and responder status with linear regression using data from T_1_. Age, gender, and race (dichotomized as black and non-black) were adjusted. To further correct for the batch effect and bias caused by unknown confounders such as cell composition, we performed surrogate variable analysis (SVA) using the R package “SVA”. Three significant SVs were detected. The associations described above were then tested again with three SVs being included in the modeling. Only CpG sites identified in both sets of analyses were selected for further examination. For array analysis, nominal significance was defined as p value≤0.05, and genome-wide significance was defined as q value ≤0.05 [26].

To verify the CpG sites identified from the array that showed responder-specific T_0_-T_1_ changes in DNAm, we assayed 33 pairs of samples with bisulfite pyrosequencing and calculated the percent of methylation. As T_0_ and T_1_ DNAm are paired by subjects, to better account for between-subject random effects, we used mixed models, in which a responder*time interaction was tested. Mixed models were also utilized to verify the CpG sites whose T_1_ DNAm showed association with response to treatment. In these verification analyses, both T_0_ and T_1_ data were used with correlations between T_0_ and T_1_ being considered. We also calculated an M value as $M=log_{2}\frac{methylation\;\%}{1-methylation\;\%}$, which was examined to ensure the validity of conclusions from models using beta values.

To determine genetic effects on DNAm, we clustered the beta values into full-, semi- and non-methylated groups by examining the distribution of the beta values. The methylation groups were then compared with the genotypes. To test the genetic association with the responder status, we used an additive model in which genotypes were numerically coded according to the number of minor allele. The genetic association was then tested using a logistic model.

To assess the relationship between gene expression and DNAm, we used the Spearman correlation. We also examined the association of *LDHC* expression with response to treatment, and the responder-specific changes between T_0_ and T_1_ in *OTX2* expression. As the expression data exhibited log-normal distribution, the associations were tested using generalized estimating equation assuming a log-normal distribution of the data. Verification analyses were conducted using SAS 9.4 unless otherwise specified. P values ≤ 0.05 were designated statistically significant.

## Results

### Study overview and population characteristics

Our discovery population consisted of 20 exacerbating asthmatics who were not exposed to nasal steroid within the past 14 days (S3 Table). To identify CpG sites whose DNAm status was associated with systemic corticosteroid treatment response, we assayed the genome-wide nasal methylation using paired DNA samples collected at both T_0_ and T_1_ from these 20 patients (40 samples total). As false positive results would occur even after stringent QC, data preprocessing, and complex analyses of 450K arrays, we further verified significant associations using bisulfite pyrosequencing in the complete cohort.

The discovery population was composed of 60% black males, and 45% were good responders. There were no statistically significant differences in age, sex, race or number of good or poor responders between the discovery population and the 13 additional subjects (S3 Table). Among the 13 additional subjects, 7 of them reported previous exposure to nasal steroid (S3 Table). Therefore, the DNAm data at T_0_ of these 7 subjects measured by bisulfite pyrosequencing were excluded from the analyses when T_0_ samples were studied. Mean baseline respiratory symptom frequency score and ACT scores were also comparable. When comparing good and poor responders in the complete cohort, we found that most of the demographics and clinical features were also similar (Table 1). While the poor responders had a slightly higher mean ACT (17.3 vs. 14.6, p = 0.047), the means of both groups are <19, indicating poor asthma control. Overall there were no major differences between the populations.

**Table 1:** Demographics and clinical features of good and poor responders.

	Good responders (N = 15)	Poor responders (N = 18)	p-value
Age	8.0 (6.0–14.0)	6.5 (5.0–11.0)	0.31
Male Sex	10 (67%)	12 (67%)	1.00
Race			
White	2 (13%)	4 (22%)	0.86
Black	12 (80%)	12 (67%)	
Biracial	1 (7%)	2 (11%)	
Exposed to nasal steroid	4 (27%)	3 (17%)	0.67
Respiratory symptom score	2.0 (1.1)	2.5 (1.1)	0.19
ACT Score	14.6 (3.5)	17.3 (3.7)	0.047

Note: age was shown as median (IQR) and tested using Wilcoxon rank sum test; respiratory symptom and asthma control scores were shown as mean (SD) and tested using t tests; the rest of the variables were shown as n (%) and tested using Fisher’s exact tests.

### Systemic steroid treatment significantly altered the nasal methylome within 24 hours in good responders, but not in poor responders

**Fig 2:**
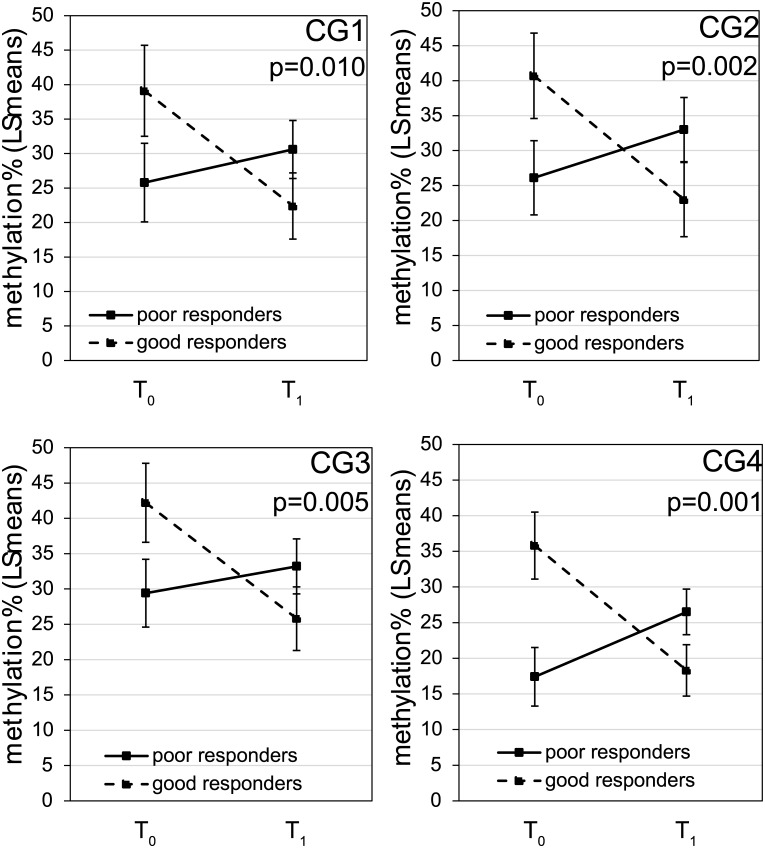
Promoter methylation of *OTX2* showed differential changes in response to steroid treatment between good and poor responders. The association of DNAm at 4 CpG sites located within the promoter region of *OTX2* with time (T_0_ vs. T_1_) and responder status were tested using mixed models with age, sex and race being adjusted. Data shown were least square means with standard errors of the methylation% at T_0_ and T_1_ for good and poor responders respectively. P values were for the responder-specific T_1_-T_0_ changes.

Using the array data, we compared the methylation level for each CpG site between T_0_ and T_1_ in good and poor responders respectively. In poor responders, no CpG sites had changes in methylation between T_0_ and T_1_ even at nominal level. In contrast, in the good responders, 5 CpG sites were identified whose methylation were significantly altered between T_0_ and T_1_ at nominal level, but did not reach genome-wide significance (p≤0.05, absolute deltaBeta≥0.10, S4 Table). Interestingly, 3 of the 5 CpG sites are in the promoter (defined as 2kb upstream TSS) and 5’ UTR of *OTX2*, suggesting a possible regulatory role for these CpG sites. As two of the three *OTX2* CpGs are adjacently located, we were able to assay them and two additional CpGs in the same region using one bisulfite pyrosequencing reaction (S1 Table). In all 4 CpG sites, significant responder*time interactions were found (Fig 2). These results confirmed that changes in DNAm from T_0_ to T_1_ differed by response status. Specifically, in good responders, DNAm levels of CpG1-4 in the *OTX2* promoter were significantly lower at T_1_, while in poor responders, the DNAm level at T_1_ was comparable to T_0_ (CpG1-3) or elevated (CpG4).

### DNAm in nasal cells separates poor responders from good responders

**Fig 3:**
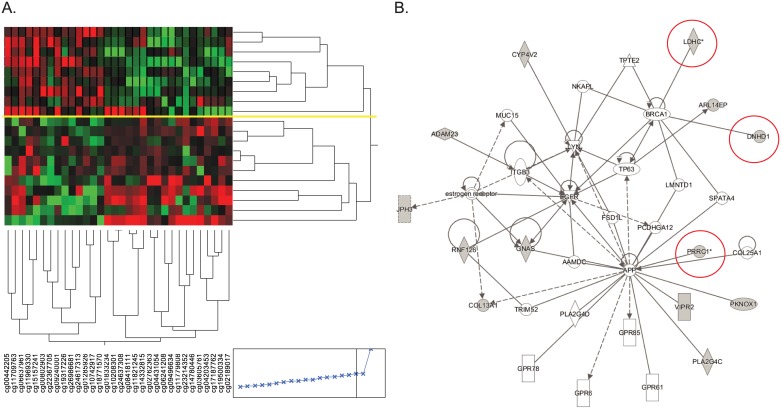
DNAm at 32 non-SNP CpG sites separate two responder groups. A. Hierarchical clustering of participants based on DNAm (Ward’s minimum variance method, JMP). Yellow line represents the complete separation between good (top panel) and poor responders (bottom panel). B. Overrepresented network from IPA analysis. CpG sites located close to genes circled in red were selected for verification.

To identify markers that distinguish poor from good responders in the discovery phase, we focused on samples collected at the T_1_ time point. In the verification phase, T_0_ samples that had base line nasal steroid usage were excluded. Using the discovery array data of the T_1_ samples (Fig 1), at nominal level, we identified 309 autosomal CpG sites with differential DNAm between the good and poor responders. Because SNPs can modify DNAm *in cis*, we further removed CpGs whose DNAm might be subject to genetic influences. Among these CpG sites, 127 have no reported common SNPs at the C or G, but 42 of these 127 sites have reported SNPs present on the array probes (<10bp away from the given CpG site) thus their DNAm measurements may not be accurate due to the influences of the SNPs (Fig 1). After removing these 42 sites, the remaining 85 sites could distinguish the two responder groups using hierarchical clustering (Ward’s minimum variance method in JMP). We then examined the distribution of the array data for these 85 sites, as well as the biological functions of genes near these CpG sites. This process resulted in 32 CpG sites whose DNAm levels were sufficient to completely separate the two responder groups (Fig 3A and S5 Table), and none of them were measured by cross-reactive probes [27]. Two CpG sites (cg17187762 and cg00802903) that reached genome-wide significance (q value <0.05, with and without SV) were included in these 32 sites.

IPA pathway analysis of the genes implicated by these 32 CpG sites identified one network that is over represented by the genes close to the 32 CpG sites (Fig 3B). Among genes in this network, *LDHC*, *Dynein heavy chain domain 1* (*DNHD1*), and *proline-rich coiled-coil 1* (*PRRC1*) were chosen for verification. *LDHC* encodes lactate dehydrogenase and is highly expressed in asthmatics. Its level in sputum has been established as a diagnostic marker for asthma [28, 29]. *DNHD1* encodes Dynein Heavy Chain Domain 1, which is a protein complex that is involved in microtubule movement and expressed in the lung. DNAm at multiple CpG sites within this gene, including the one identified from our study (cg10208301), is associated with multiple sclerosis in blood DNA [30]. The proline-rich coiled-coil 1 protein encoded by *PRRC1* is highly expressed in oral epithelium and moderately expressed in lung tissues. Three CpG sites within the gene body of *DNHD1* (including cg10208301), three CpG sites within the promoter of *LDHC* (including cg11821245), and three CpG sites within the promoter of *PRRC1* (including cg02762363 and cg04431054) were measured by bisulfite pyrosequencing in both T_0_ and T_1_ samples from all 33 participants and their association with length of stay was examined. As shown in Table 2, none of the sites showed differences in DNAm between T_0_ and T_1_, therefore data from T_1_ and T_0_ were combined and compared between good and poor responders. Using this approach, all CpG sites identified by the arrays were verified, in which good responders exhibited lower methylation compared to poor responders (Table 2). In addition, two CpG sites in *LDHC* that were not evaluated by the array also exhibited significant differences between good and poor responders. Therefore, we identified novel epigenetic biomarkers that may not be sensitive to the steroid treatment, but can differentiate poor responders from good responders.

**Table 2:** CpG sites whose DNAm levels at T1 are associated with treatment response.

Gene	site	Target ID	T_1_			T_0_			T_1_ + T_0_		
			poor	good	p-value	poor	good	p-value	poor	good	p-value
DNHD1	CG1	cg10208301	92.7±4.2	83.3±4.8	0.13	93.7±3.4	83.9±3.9	0.06	93.1±2.3	84.0±2.7	**0.011**
	CG2		84.0±3.8	76.1±4.1	0.17	85.8±2.8	80.1±3.3	0.20	85.3±2.1	79.6±2.3	0.08
	CG3		90.5±5.3	81.0±6.1	0.22	89.5±3.6	87.7±4.1	0.73	89.0±2.8	87.0±3.2	0.64
LDHC	CG1		81.0±3.8	72.1±4.3	0.11	87.7±2.1	78.6±2.4	**0.007**	85.5±1.8	77.3±2.1	**0.004**
	CG2		74.9±4.3	61.8±4.9	**0.040**	79.3±3.8	70.2±4.3	0.11	77.3±2.6	67.5±2.9	**0.012**
	CG3	cg14332815	67.6±4.2	53.8±4.8	**0.028**	67.5±4.7	57.6±5.4	0.16	67.4±2.6	55.8±3.0	**0.004**
PRRC1	CG1		37.8±3.8	32.0±4.3	0.30	32.8±4.5	41.0±5.1	0.21	35.0±3.0	36.2±3.4	0.76
	CG2	cg02762363	26.0±2.2	19.7±2.5	**0.05**	28.4±5.1	22.2±5.7	0.40	27.1±2.4	20.4±2.7	0.06
	CG3	cg04431054	32.7±2.5	24.5±2.8	**0.026**	38.8±4.8	27.4±5.5	0.11	34.5±2.6	24.9±2.9	**0.014**

Note: DNAm were compared between good and poor responders using mixed models with age, sex and race being adjusted. Data shown were least square means and standard errors.

### DNAm levels in candidate genes correlate with gene expression

**Fig 4:**
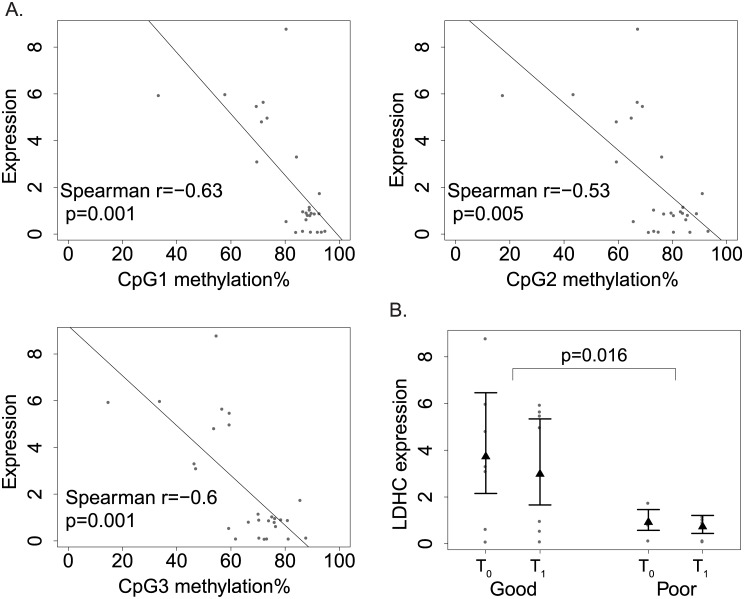
Relationships between *LDHC* expression, DNAm and treatment response. A. Correlations between *LDHC* expression and DNAm at each of the three CpG sites. Paired methylation% and expression values were plotted for each sample as dots; the regression lines showed negative correlation between expression and DNAm. B. Differential *LDHC* expression between good and poor responders. The expression data were plotted as dots; the least square mean of each group was shown as solid triangle along with 95% confidence limits.

To assess the role of DNAm in the regulation of gene expression, we assayed the expression levels of *OTX2*, *LDHC*, *DNHD1*, and *PRRC1* in the same samples where DNAm levels were measured. In *OTX2*, moderate negative correlation was detected between expression and DNAm but did not reach statistical significance (Spearman r = -0.3 for all sites, p value ranging from 0.08–0.12, S1A Fig). In *LDHC*, we observed strong negative correlation between expression and DNAm of all three CpG sites (Fig 4A, Spearman r ranging -0.6−-0.5, p<0.01 for all sites). No significant negative correlations between expression and DNAm were found for *DNHD1* and *PRRC1* (data not shown). Moreover, significant association between *LDHC* expression and response to treatment was detected (Fig 4B, p = 0.016). Consistent with their lower promoter DNAm in *LDHC*, good responders showed higher *LDHC* expression, supporting the down-regulation of *LDHC* expression by promoter DNAm. In *OTX2*, responder-specific T_1_-T_0_ changes in expression was suggested, but did not reach statistical significance (S1B Fig, p = 0.06). In conclusion, we observed negative correlation between promoter DNAm and expression for *LDHC* and *OTX2*, but not for *DNHD1* and *PRRC1*.

### Genetic variation modifies DNAm and associates with treatment response

**Fig 5:**
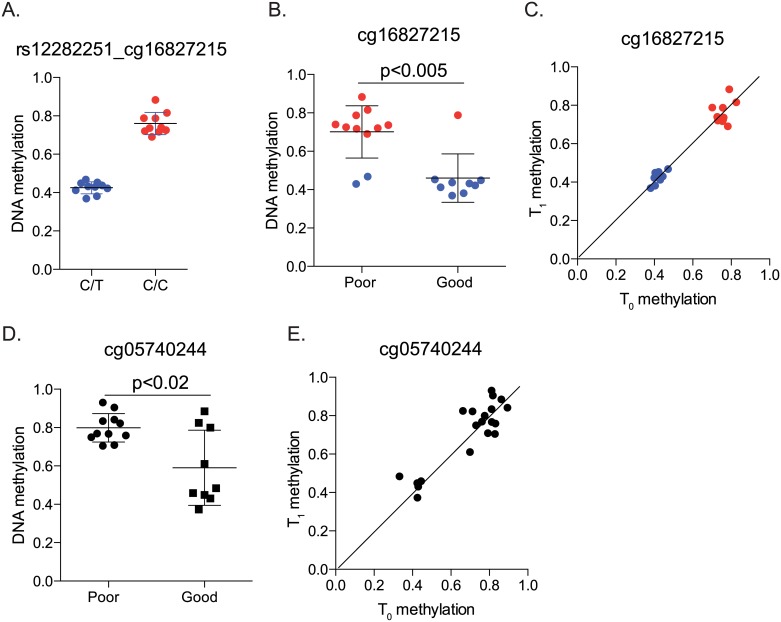
Relationships between DNAm, genotype at meSNPs and treatment response. A-B. Relationship between rs12282251, cg16827215 and treatment response. Red and blue dots represented the CC and CT genotypes, respectively. C. Good agreement of DNAm at cg16827215 between T_0_ and T_1_. D. DNAm at cg05740244 between good and poor responders. All participants have the same genotype (GG). E. Good agreement of DNAm at cg05740244 between T_0_ and T_1_. Identity lines were drawn in C and E.

Among the 309 sites whose DNAm levels were associated with length of stay and therefore differentiated poor responders from good responders, 182 had SNPs located at C or G according to the 1000 genomes project (S6 Table). Such SNPs have the ability to disrupt or create a CpG dinucleotide and thereby substantially modify DNAm. To examine the potential effects of SNPs on DNAm, we visualized the distribution of DNAm levels by genotypes of 7 SNPs. At six sites, a tri-modal distribution of DNAm was observed which completely aligned with the genotypes (Table 3). Given the significant associations between DNAm and treatment response and the good alignment between DNAm and genotypes, it is not surprising that in the 20 subjects who had 450K array data, all the sites showed statistically significant or marginally significant genetic associations with responder status. For example, rs12282251 (C/T) is located at the C position of cg16827215. The methylation levels at cg16827215 were completely aligned with the genotypes (Fig 5A), with CC (red dots) showing complete methylation and CT (blue dots) showing semi-methylation. Association of cg16827215 DNAm and genetic association of rs12282251 with the responder status were also detected (Fig 5B and Table 3). When all 33 genotyped subjects were analyzed using additive models, similar genetic associations were observed (Table 3); significant associations remained for four sites (rs12282251, rs66630638, rs73006518 and rs76828276) while the other two (rs34756202 and rs73006518) showed marginal significance. For rs75428195, although there is reported G/C variation in the general population, we did not detect the C allele in our cohort, and all subjects had a GG genotype. Therefore, the observed full and semi-methylation at cg05740244 (Fig 5D and 5E) was not due to genetic variation, and responder status was only associated with methylation levels, but not genotypes (Fig 5D and S7 Table). Moreover, similar to sites shown in Table 2, these two sites also exhibited good DNAm agreement between T_0_ and T_1_ (Fig 5C and 5E). As T_0_ samples are not always available, our results therefore implicate the potential usage of T_1_ DNAm as a marker to differentiate good from poor responders.

**Table 3:** Associations between DNAm, genotypes at meSNPs, and treatment response.

rsID	cg	Genotype (n)	beta at T_1_ (SD)	%Poor responders	Genetic association (p value)	Chr	location	MA (%) (ED)	MA (%) (UCSC hg19)	Putative function
rs12282251	cg16827215	CC (20)	0.76 (0.06)	75%	0.003	11	11292635	T (0.20)	T (0.18)	3’ UTR
		CT (13)	0.43 (0.03)	23%						
rs34756202	cg01882774	CC (17)	0.83 (0.03)	35%	0.11	14	94491763	T (0.30)	T (0.26)	Intron/upstream
		CT (12)	0.50 (0.10)	83%						
		TT (4)	0.07 (0.03)	50%						
rs7933565	cg19680693	GG (12)	0.83 (0.04)	42%	0.10	11	94111808	A (0.45)	A (0.53)	3’ UTR
		GA (12)	0.52 (0.07)	50%						
		AA (9)	0.11 (0.05)	78%						
rs66630638	cg08891829	GG (22)	0.93 (0.01)	36%	0.002	4	56231728	A (0.16)	A (0.14)	intron
		AG (10)	0.52 (0.05)	90%						
		AA (1)	0.04 (-)	100%						
rs73006518	cg22843488	CC (22)	0.90 (0.02)	41%	0.015	3	151017685	T (0.19)	T (0.06)	intron
		CT (9)	0.67 (0.10)	78%						
		TT (2)	0.13 (0.02)	100%						
rs76828276	cg05187322	CC (24)	0.90 (0.02)	42%	0.010	17	78152328	T (0.12)	T (0.07)	5’ UTR
		CT (9)	0.49 (0.04)	89%						

Note: Minor allele and frequencies from current ED study and UCSC (hg19) were listed. For the genetic association tests, additive models were used. No effects of age, sex or race on the responder status were detected.

## Discussion

In this pilot study, we utilized a genome-wide approach to first search for CpG sites whose DNAm levels respond to steroid treatment for asthma exacerbation in nasal epithelial cells. We found that the promoter of *OTX2* showed significantly decreased methylation after treatment, but such decrease was only observed in children who responded well, not in children who responded poorly. Using the same data, we also identified a novel set of CpG sites whose methylation are unresponsive to treatment, yet distinguish the poor responders from the good responders. Interestingly, quite a few of these CpG sites can be disrupted by the presence of certain common SNPs; in most of these sites, DNA methylation was altered along with their genotypes. Taken together, our findings suggest that epigenetic mechanisms underlie the heterogeneous responses to systemic steroid treatment, in part driven by genetic variations present at CpG sites.

The *OTX2* gene encodes a transcription factor with key roles in the brain [31, 32], craniofacial and sensory organs [33, 34] and pituitary development [35]. Upon differentiation of pluripotent stem cells, it also interacts with Oct4 to activate gene enhancers by establishing and maintaining local active chromatin [36]. The role of *OTX2* in asthma and response to steroid treatment is unknown. Interestingly, Otx2 can directly interact with Foxa2 [37, 38], and such interactions can be detected *in vivo* and govern development of the anterior mesendoderm, node, and midline in mice [38]. Foxa2 has been implicated in suppression of goblet cell metaplasia during allergen challenge [39]. It suppresses the production of IL13, IL33, CCL20 and CCL17 from airway epithelial cells and inhibits allergen-induced goblet cell differentiation [40], through reprogramming of Th2-mediated inflammation and innate immunity. Whether *OTX2* has similar roles in response to allergen warrants further mechanistic studies. We also found no dexamethasone responsive elements or GR binding sites within 5000 bases of *OTX2* in A549 cells treated with dexamethasone in a publically available GR ChIP-seq dataset [41]. No significant changes in expression or methylation of *OTX2* were detected after dexamethasone treatment (24hrs) in human bronchial airway epithelial cells (data not shown). However, in nasal samples from children, as opposed to poor responders who showed no significant changes in DNAm and expression of *OTX2*, good responders showed decreased promoter methylation after steroid treatment (Fig 2) and a trend of increase in expression (S1 Fig).

We have also identified 309 CpG sites whose DNAm levels were associated with treatment response—182 overlap with common SNPs and 127 do not have SNP co-localization. Out of the CpGs without common SNP co-localization, we followed up on sites located at *LDHC*, *DNHD1* and *PRRC1*, and verified the associations of their DNAm with the treatment response. Moreover, we also detected significant differential expression between the two responder groups in *LDHC* (Fig 4B). The level of lactate dehydrogenase level in sputum has been established as a diagnostic marker for asthma [28, 29], and our data suggest that it might also be a useful marker for steroid treatment response. Among all verified sites, no CpG sites were found to be strongly associated with poorly controlled asthma (ACT score), or base line asthma symptom scores.

Out of the 182 SNP-containing sites, we genotyped 7 and found complete agreement between genotypes and DNAm levels at 6 sites (Table 3). Furthermore, four sites showed statistically significant genetic association with the treatment response. These four sites are located within *GALNTL4 (or GALNT18*, Polypeptide N-Acetylgalactosaminyltransferase 18), *SRD5A3* (Steroid 5 Alpha-Reductase 3), *MED12L-GPR87* locus (mediator complex subunit 12-like and G protein-coupled receptor 87) and *CARD14* (Caspase Recruitment Domain Family Member 14). *GALNTL4* encodes a glycosyltransferase that participates in the initial steps of protein O-glycosylation and it is involved in the biosynthesis of simple mucin-type carbohydrate antigens and responsive to bacterial infection in mice gastric mucosa [42]. *SRD5A3* encodes steroid 5-alpha reductase, plays a key role in the early steps of protein N-linked glycosylation [43] and is required for the production of 5-alpha-dihydrotestosterone (DHT) from testosterone in hormone-refractory prostate cancer [44]. rs73006518 is located within two protein coding genes, *MED12L* on the + strand and *GPR87* on the—strand. The protein encoded by *MED12L* exhibits sequence similarity with components of the Mediator complex and may be involved in gene transcription. The G protein receptor encoded by *GPR87* is a receptor for lysophosphatidic acid (LPA) [45], and LPA may contribute to asthma by regulation of cytokine synthesis and chemotaxis in lymphocytes [46–49], contractility and proliferation of smooth muscle cells [50], and inflammatory signaling in airway epithelium [51]. LPA is also required for p53-dependent cell survival in response to DNA damage [52] and its overexpression leads to lung tumor cell proliferation [53]. The protein encoded by *CARD14* belongs to the membrane associated guanylate kinase (MAGUK) family, and may function as an upstream activator of BCL10 and NF-kappaB signaling and contribute to apoptosis [54]. Its expression in bronchial airway epithelial cell was associated with exhaled nitric oxide in severe asthmatics [55]. Although these studies support the biological functions of these genes in asthma phenotypes, the potential utilization of these biomarkers to sufficiently predict future treatment response in children hospitalized for asthma exacerbation awaits further evaluation in longitudinal studies studying a large number of children in multiple hospitalizations. Once established, these biomarkers will inform clinicians about a patient’s treatment response to steroid during future asthma attacks and identify those who will be in need of alternative individualized asthma management plan. This would significantly reduce length of hospitalization and improve patient care.

It is intriguing that the genotype at C or G sites can modify DNAm levels *in cis* in airway epithelial cells, and when C is present, it is always methylated. This indicates the complete dependence of DNAm on genetic sequence at these locations, and is consistent with previous reports that in CD4^+^ T cells over 80% of meSNPs are meQTLs [16]. The dictation of DNAm by so-called small methylation determining regions has been observed during stem cell differentiation [56]. Our findings also indicate that DNAm and genotypes can infer each other very well at some genomic locations (6 out of 7 that we genotyped). Though it is difficult to tease out which is truly the determinant for phenotype in our association study, because of its role in gene expression regulation, DNAm becomes an intriguing explanation for the biological effects of these genetic variants, especially those located in regulatory regions. Detailed mechanisms accounting for such tight regulation of DNAm by meSNPs warrants further investigation. In addition, we also found one case where genotype did not completely follow levels of DNAm. Therefore, genetic and epigenetic studies in the same population are both required to fully understand the interplay between these two mechanisms and their collaborative contribution to biological and pathological processes.

## Conclusions

Despite small sample size, our research showed that DNAm variations can differentiate good responders and poor responders. These epigenetic biomarkers may be used to distinguish children who do not respond to steroid treatment well, therefore are in need of alternative novel therapies. Further replication studies in children will be needed to substantiate these finding, and the establishment of such biomarkers of treatment response will inform personalized management plan during severe pediatric asthma exacerbations. DNAm biomarkers are more interactive with environment, especially those independent of genetics. They reflect real time dynamics of a patient, thus may better predict responses to asthma treatment from episode to episode. Our findings in the genetic-dependent DNAm variation provide new insights for phenotypic variations associated with genetics. Besides being used as biomarkers, DNAm can also be targets for future novel alternative therapies.

## Supporting information

S1 Fig Relationships of *OTX2* expression with DNAm and treatment response. A. Correlations between *OTX2* expression and DNAm at each of the four CpG sites. Paired methylation% and expression were plotted for each of the samples as dots; the regression lines suggested negative correlation between expression and DNAm. B. *OTX2* expression for good and poor responders at different time points. The expression data were plotted as dots; the least square mean of each of the groups was shown as solid triangle along with 95% confidence limits. Responder-specific T_1_-T_0_ change in expression was suggested (p = 0.06).
(EPS)

S1 Table Primers used for pyrosequencing and the CpG and SNP sites interrogated. (XLSX)

S2 Table Primers used for RT-qPCR. (DOCX)

S3 Table Demographics of the patients included in the discovery phase and verification phase. (DOCX)

S4 Table List of CpG sites with methylation changes from T_0_ to T_1_ in good responders. (XLSX)

S5 Table List of non-SNP CpG sites that showed differential methylation levels between good responders and poor responders. (XLSX)

S6 Table List of SNP-CpG sites that showed differential methylation levels between good responders and poor responders. (XLSX)

S7 Table Associations of treatment response with rs75428195 genotype and DNAm at cg05740244. (DOCX)

## Abbreviations

ACT: Asthma control test
DNAm: DNA methylation
ED: Emergency department
ICS: Inhaled corticosteroid
IPA: Ingenuity pathway analysis
LOS: Length of stay
MAF: Minor allele frequency
meQTL: Methylation quantitative trait locus
SNP: Single nucleotide polymorphism
SVA: Surrogate variable analysis
