# Supplementary material for: Nasal DNA methylation differentiates corticosteroid treatment response in pediatric asthma: A pilot study
Source: PLoS One. 2017 Oct 13;12(10):e0186150. doi: 10.1371/journal.pone.0186150 (PMC5640236; doi:10.1371/journal.pone.0186150)
Supplement: S2 Table — (DOCX) [file pone.0186150.s003.docx]

**S2 Table. Primers used for RT-qPCR**

| **Gene** | **Forward** | **Reverse** |
| --- | --- | --- |
| OTX2 | 5'-GAGGTGGCACTGAAAATCAAC-3' | 5'-TCTTCTTTTTGGCAGGTCTCA-3' |
| LDHC | 5’-AAGCTGCCATGGTTGGATTA-3’ | 5’-AGGGTCCAGAGTCTTCAGAGC-3’ |
| DNDH1 | 5’-CCCTGTACGGTATGAAAGCAG-3’ | 5’-AGGAAGTCTCAGGTGAAAAGC-3’ |
| PRRC1 | 5’-GCTATGTCTTCTACCCCTGTTC-3’ | 5’-CAAAAGGTAATGGTGCTGAAGG-3; |
| GAPDH | 5'-GGGGAAGGTGAAGGTCGGAGTCA-3' | 5'-AGCCTTGACGGTGCCATGGAAT-3' |
