# Supplementary material for: Nasal DNA methylation differentiates corticosteroid treatment response in pediatric asthma: A pilot study
Source: PLoS One. 2017 Oct 13;12(10):e0186150. doi: 10.1371/journal.pone.0186150 (PMC5640236; doi:10.1371/journal.pone.0186150)
Supplement: S3 Table — (DOCX) [file pone.0186150.s004.docx]

**S3 Table. Demographics of the patients included in the discovery phase and verification phase.**

|  | | | |
| --- | --- | --- | --- |
|  | Discovery  (N=20) | Additional participants  (N=13) | p-value |
| Age | 10.0 (6.0-15.0) | 6.0 (5.0-8.0) | 0.09 |
| Male sex | 16 (80%) | 6 (46%) | 0.06 |
| Race |  |  |  |
| white | 4 (20%) | 2 (15%) | 1.00 |
| black | 14 (70%) | 10 (77%) |  |
| biracial | 2 (10%) | 1 (8%) |  |
| Response to corticosteroids |  |  |  |
| good | 9 (45%) | 6 (46%) | 1.00 |
| bad | 11 (55%) | 7 (54%) |  |
| Exposed to nasal steroid | 0 (0%) | 7 (54%) | <0.001 |
| Respiratory symptom score | 2.2 (1.3) | 2.4 (0.7) | 0.57 |
| ACT score | 15.8 (3.7) | 16.5 (4.3) | 0.63 |

Note: age was shown as median (IQR) and tested using Wilcoxon rank sum test; respiratory symptom and asthma control scores were shown as mean (SD) and tested using t tests; the rest of the variables were shown as n (%) and tested using Fisher’s exact tests.
