# Supplementary material for: Nasal DNA methylation differentiates corticosteroid treatment response in pediatric asthma: A pilot study
Source: PLoS One. 2017 Oct 13;12(10):e0186150. doi: 10.1371/journal.pone.0186150 (PMC5640236; doi:10.1371/journal.pone.0186150)
Supplement: S7 Table — (DOCX) [file pone.0186150.s008.docx]

**S7 Table. Associations of treatment response with rs75428195 genotype and DNAm at cg05740244.**

|  |  | Bad responder | Good responder | P value |
| --- | --- | --- | --- | --- |
| Genotype | GG | 11 (90%) | 9 (10%) | - |
| Mean beta at T_1_ (SD) |  | 0.80 (0.07) | 0.59 (0.20) | 0.013 |
| Methylation group | Full | 11 (100%) | 4 (44%) | 0.008 |
|  | semi | 0 (0%) | 5 (56%) |  |

Note: beta values were compared between good and bad responders using t test; the proportion of methylation groups between good and bad responders were compared using Fisher’s exact test.
